# Optical Coherence Tomographic Findings in Highly Myopic Eyes

**Published:** 2010-04

**Authors:** Hooshang Faghihi, Fedra Hajizadeh, Mohammad Riazi-Esfahani

**Affiliations:** 1 Noor Ophthalmology Research Center, Noor Eye Hospital, Tehran, Iran; 2 Farabi Eye Hospital, Tehran University of Medical Sciences, Tehran, Iran

**Keywords:** Optical Coherence Tomography, OCT, High Myopia

## Abstract

Optical coherence tomography (OCT) has enhanced our understanding of changes in different ocular layers when axial myopia progresses and the globe is stretched. These findings consist of dehiscence of retinal layers known as retinoschisis, paravascular inner retinal cleavage, cysts and lamellar holes, peripapillary intrachoroidal cavitation, tractional internal limiting membrane detachment, macular holes (lamellar and full thickness), posterior retinal detachment, and choroidal neovascular membranes. In this review, recent observations regarding retinal changes in highly myopic eyes explored by OCT are described to highlight structural findings that cannot be diagnosed by simple ophthalmoscopy.

## INTRODUCTION

Pathologic or high myopia is defined as a refractive error of −6.00 diopters (D) or greater and an axial length exceeding 26 mm.^1^ Fundus changes in pathologic myopia are numerous and related to the degree of myopia, axial length of the globe, presence of a posterior staphyloma, and age. The scleral thinning accompanied by expansion of the globe leads to formation of a posterior staphyloma and optic disc myopic conus.^2^

Optical coherence tomography (OCT) has recently made it possible to explore changes in ocular layers as axial myopia progresses and the globe is stretched. These findings consist of dehiscence of retinal layers known as retinoschisis, paravascular inner retinal cysts and lamellar holes, peripapillary intrachoroidal cavitation (also known as peripapillary detachment in pathologic myopia), tractional internal limiting membrane detachment, macular holes (lamellar and full thickness), posterior retinal detachment and choroidal neovascular membranes.

In this review, we describe retinal changes in highly myopic eyes according to findings observed by OCT.

## DEFINITION

The early stages of myopic retinal changes can easily be underestimated by biomicroscopy, angiography or ultrasonography, and therefore remain undiagnosed. Recently, OCT with its fine cross-sectional imagery of retinal structures has greatly facilitated the evaluation of posterior vitreoretinal anatomy in eyes with high myopia. It can also reveal otherwise undetectable retinal changes in asymptomatic patients. The OCT ophthalmoscope produces longitudinal retinal B-scans and coronal C-scans.^3^ Therefore, it provides information not readily available by conventional imaging techniques or fundus examination.

### En face OCT or C-Scan

B-scan produces cross-sectional images of retinal morphology which bears strong resemblance to histology. OCT C-scans are represented as 2-dimensional transverse slices at any given depth through the retina, thereby enabling visualization of the lateral extent of different structures. OCT C-scans are associated pixel-to-pixel points with simultaneously confocal images used to identify the exact position of lesions on the posterior pole. With en face OCT, localization and relation of retinal lesions can be precisely defined, especially in myopic eyes. En face OCT provides accurate imaging of retinal abnormalities in high myopia and allows width measurement and point-to-point localization of changes.^4^ It is a noninvasive means of detecting minimal changes during follow-up.

**Figure 1:**
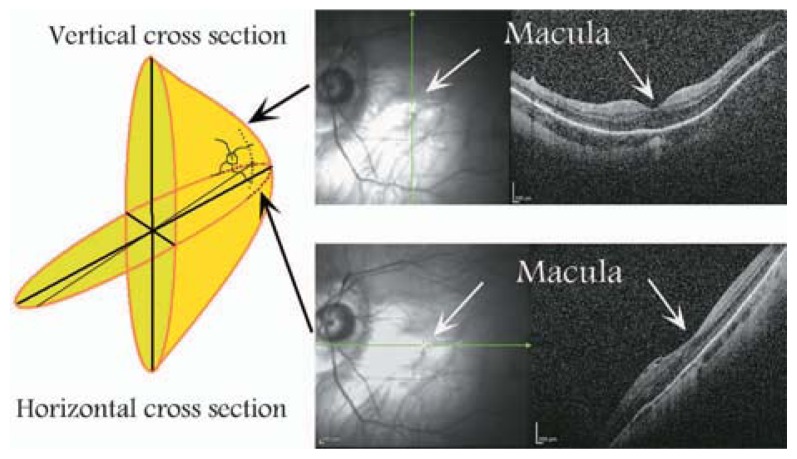
Difference in vertical and horizontal OCT cross-sections in a myopic eye with posterior staphyloma.

**Figure 2:**
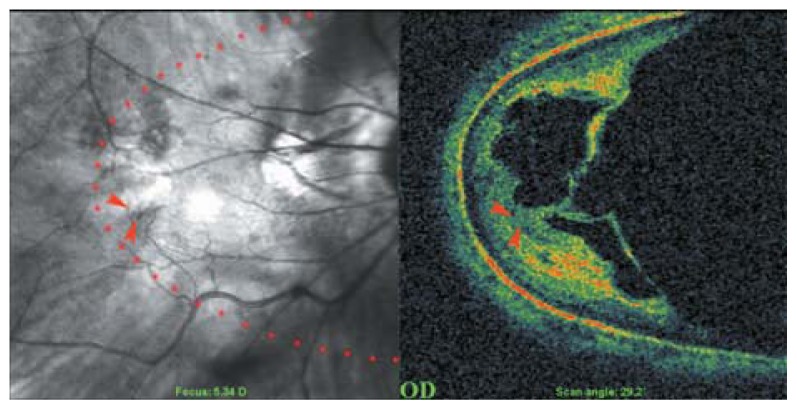
C-scan optical coherence tomography in a myopic eye shows incomplete detachment of the posterior cortical vitreous with persistent attachment and traction on the temporal part of the macula (red arrowheads). The red dots show the area of the C-scan.

**Figure 3:**
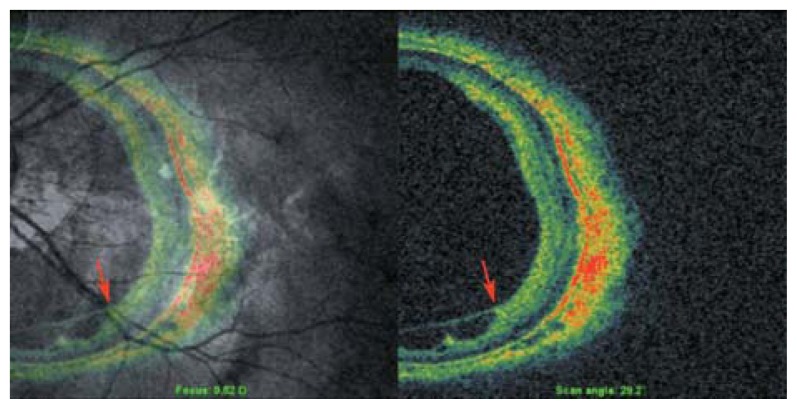
Rigid and taut retinal vessels (red arrows) may contribute to creation of retinal schisis in myopic eyes.

Myopic posterior staphyloma, with its outward globe bulge, results in a deep concave B-scan OCT and distorted retinal structures. Because of the smaller base curvature of the horizontal section and larger base curvature of vertical section, macular structures can be defined more easily by vertical sections (especially when the macula lies in the slope of the staphyloma, Fig. 1). Due to structural distortion and posterior staphyloma formation in myopic eyes, the C-scan mode can define retinal layers more precisely than longitudinal B-scan (Figures 2, 3). En face OCT-assisted surgery in myopic eyes with macular hole can facilitate the removal of premacular tractional structures.^4^

**Figure 4:**
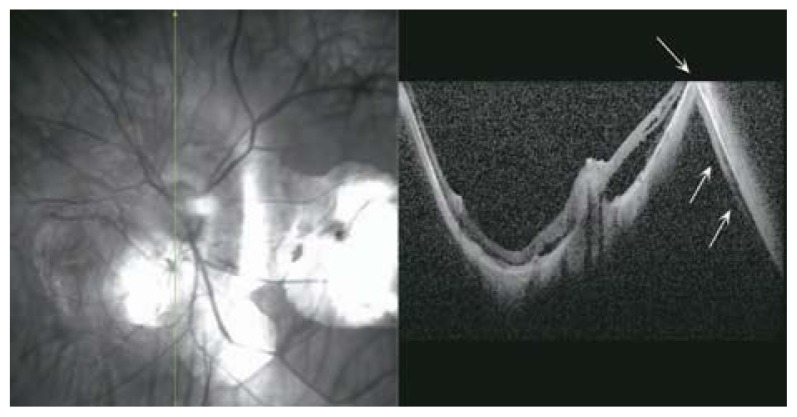
Mirror-image artifact in a highly myopic eye. The deeper the staphyloma, the more the distortion caused by such artifacts on cross-sectional images.

Another problem with OCT images obtained in myopic eyes is the mirror–image artifact. This artifact is due to a complex conjugate ambiguity of Fourier-transform of real-value spectral data.^5^ In practice, the measured sample has to be positioned at one side of the zero optical path difference position to avoid overlapping mirror images. In myopic eyes with a deep staphyloma, this artifact adversely affects images such that in one section, half of the image is upright and the other half is inverted (Fig. 4).

## CLINICAL FEATURES

### Peripapillary Intrachoroidal Cavitation

**Figure 5:**
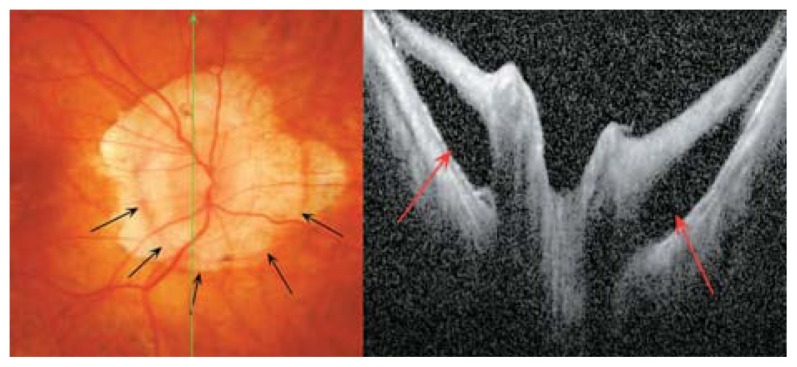
Changes in peripapillary detachment in pathologic myopia (PDPM) consist of a yellow-orange lesion in the periphery of the myopic crescent (black arrows). Cross-sectional optical coherence tomography shows a longitudinal scan of the same patient which clearly identifies hollow spaces adjacent to the disc.

Freund and associates^6^ first described this condition as peripapillary detachment in pathologic myopia (PDPM). This anomaly has been identified in 4.9% to 9.4% of highly myopic persons.^4^,^7^,^8^ PDPM is predominantly located adjacent to the inferior edge of the optic disc but in some patients it surrounds the entire optic disc. PDPM appears ophthalmoscopically as a yellowish-orange lesion around the optic disc (Fig. 5). Glaucomatous visual field defects are often detected in eyes with PDPM (70–71%).^4^,^7^,^9^ A strong correlation has been reported between the thickness of PDPM and associated visual field defects.^4^ Previously, PDPM was thought to be a localized detachment of the retinal pigmented epithelium (RPE) and retina, but OCT examination has not revealed the usual findings observed with pigmented epithelial detachment (PED). Toranzo et al^10^ referred to this condition as peripapillary intrachoroidal cavitation, because the changes did not exhibit the usual features of PED on fluorescein angiography. Early localized hyperfluorescence with progressive dye pooling due to PED is visualized as a well circumscribed dome-shaped elevation on OCT, but in PDPM the RPE looks completely normal and flat, however, deep hyporeflectivity is present in the underlying choroid, resembling intrachoroidal cavitations separating the RPE from the sclera. Hyporeflective spaces are seen in some patients only posterior to the myopic conus and the yellow-orange lesion is not seen ophthalmoscopically in these patients.

**Figure 6:**
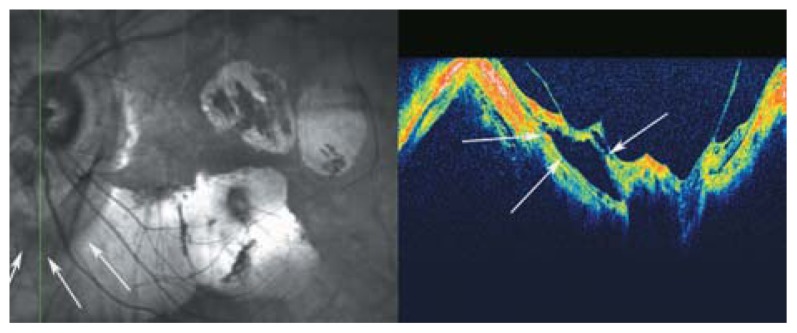
Peripapillary detachment in pathologic myopia localized to the inferior part of the optic disc (arrows). Incomplete posterior vitreous detachment with attachment of the posterior cortical vitreous to the optic disc head and paravascular cysts are also noted. The mirror image artifact resulted in a segmentally inverted image.

**Figure 7:**
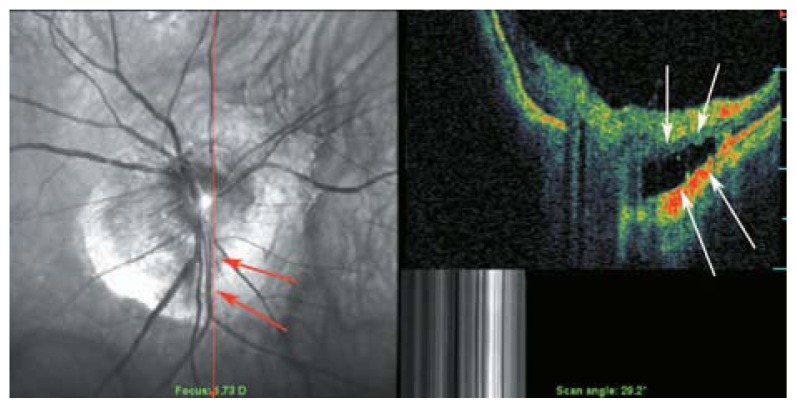
A SLO-OCT view of the optic nerve head in a myopic eye. Peripapillary cavitation is a hollow, dark space in the inferior part of optic nerve head (white arrow on OCT and red arrows on SLO sections of the picture).

**Figure 8:**
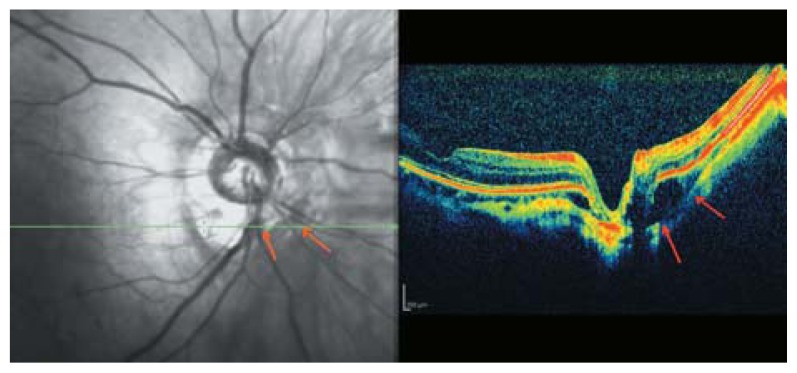
A myopic person with small peripapillary choroidal cavitation adjacent to the optic disc.

**Figure 9:**
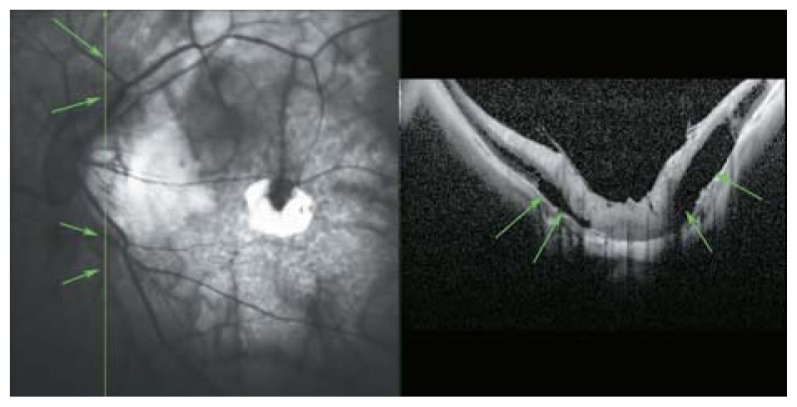
Peripapillary detachment in pathologic myopia represents as hollow spaces beside the root of the optic disc (green arrows).

The pathogenesis of PDPM is not clear, but vitreous fluid may be the source of disruption of the choroid and fluid accumulation.^11^ Because of its predominant location inferior to the optic nerve in their study, Freund et al^6^ suggested that PDPM is an incomplete form of choroidal coloboma, However, Toranzo et al^10^ hypothesized that progression of peripapillary staphyloma may result in separation of the sclera from the RPE due to cavitation within the choroid (Figures 6–9). It is possible that progression of the staphyloma separates the connection of the choroid to the optic nerve (named collagenous limiting tissue of Elschning^12^) resulting in retraction of the choroid from the optic nerve margins.^10^ Abnormalities of retinal vessels were frequently (83.9% of eyes with PDPM) detected in a study by Shimada et al.^7^ The inferotemporal retinal vein was markedly bent between the myopic conus and PDPM.

### Paravascular Retinal Cysts

**Figure 10:**
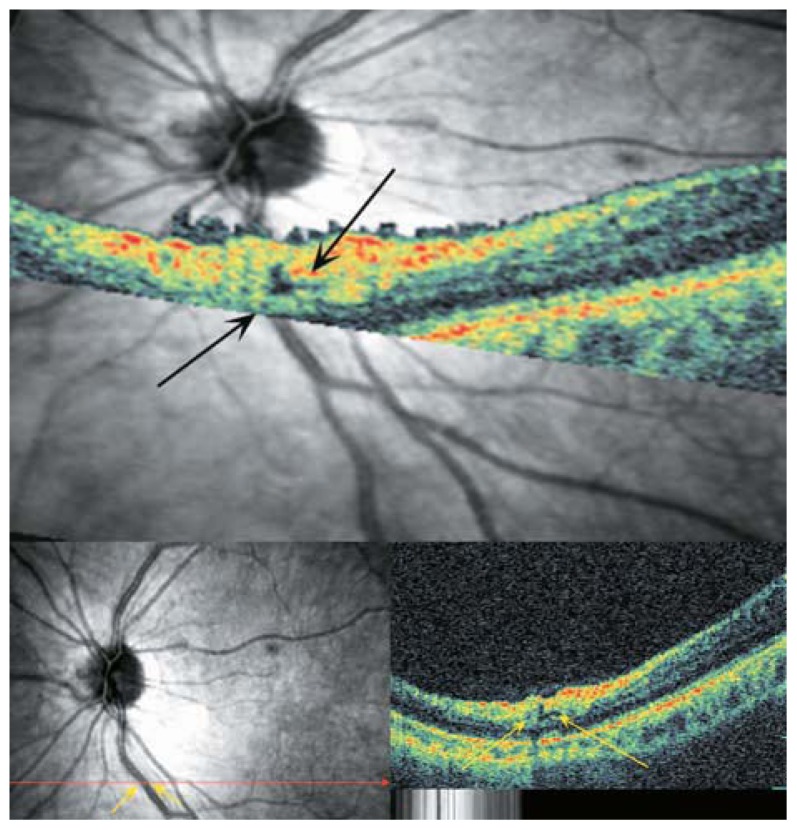
Typical small paravascular cyst located adjacent to major retinal vessels deep in the retina without communication with the vitreous cavity.

**Figure 11:**
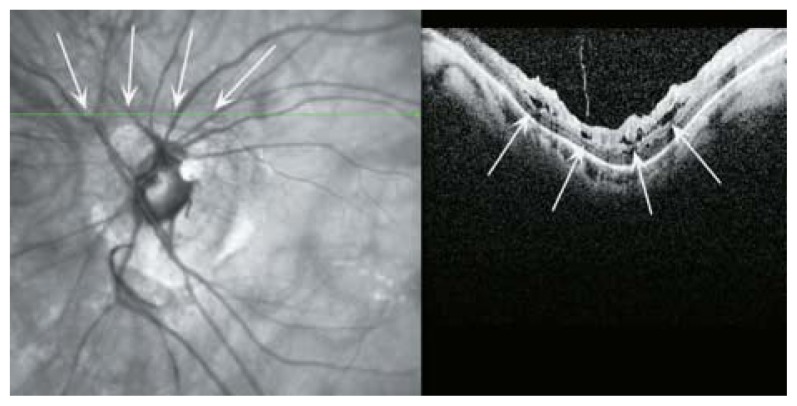
Longitudinal optical coherence tomography shows multiple paravascular cysts adjacent to main retinal vessels.

Paravascular retinal cysts were originally described histopathologically as retinal rarefaction around retinal vasculature in autopsied eyes.^13^ On OCT they appear as small hollow spaces mainly around large retinal vessels. In one study^14^ on high myopic persons with spherical equivalent myopia exceeding −8.00 D, OCT revealed retinal cysts in 49.5% of highly myopic eyes, whereas its incidence by ophthalmoscopy was only 24.4%. In a study by Forte et al^4^ on highly myopic eyes with spherical equivalent higher than −6.00 D, paravascular cysts were observed in 1.5% of cases; cysts detected by OCT measured larger than those detected by ophthalmoscopy. These findings indicate that OCT is able to detect more paravascular retinal cysts than ophthalmoscopy (Figures 10, 11). Patients with paravascular retinal cysts were significantly older, had higher myopia, longer axial length, and a higher incidence of posterior staphyloma as compared to those without retinal cysts.^14^ This demographic pattern may help explain the difference in the incidence of such cysts in the above–mentioned studies.

### Vascular Microfolds

Vascular microfolds are common in highly myopic eyes. The incidence of vascular microfolds was 2.9% in an early report^15^, but in recent studies and with advances in OCT systems, the incidence has risen to 20–44.6%.^4^,^14^ Sayanagi et al^15^ suggested that inflexibility of retinal vessels in highly myopic eyes can cause traction by the vitreous body on the retina, leading to development of vascular microfolds. Patients with both cysts and vascular microfolds had a higher incidence of a posterior staphyloma (84.3%) as compared to those with cysts alone (50.0%).^14^ Vascular microfolds may be a critical trigger for recurrent retinal detachment in patients with myopic macular holes.^16^ Vitreoretinal adhesions adjacent to retinal vessels might be the cause of microfolds and cysts.^15^

**Figure 12:**
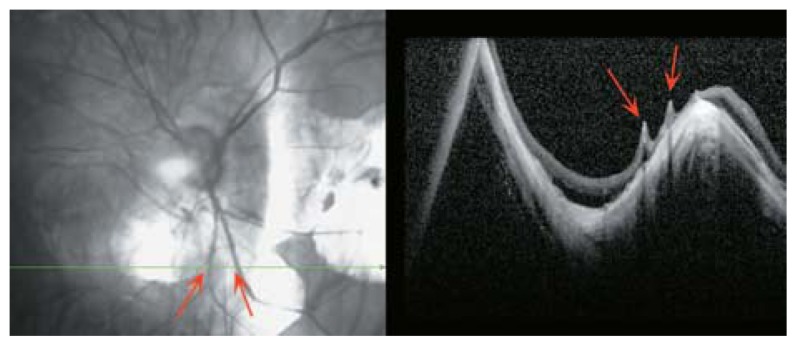
Two vascular microfolds are noted near the optic disc along large retinal vessels. Irregular staphyloma results in a bizarre shaped longitudinal optical coherence tomography image. Small extension of peripapillary detachment with a hollow space at the base of the staphyloma is also noted.

Patients with vascular microfolds and paravascular lamellar holes with paravascular retinal cysts and patients with vascular microfolds or lamellar holes were significantly older than subjects with cysts only. This suggests that paravascular retinal cysts might be the earliest indicators of traction on and around retinal vessels in highly myopic eyes (Fig. 12).^14^

In some eyes, especially those with microfolds, retinal cysts extend beyond the retinal vasculature. The formation of vascular microfolds might facilitate splitting of the retina and cause expansion of paravascular retinal cysts horizontally or vertically which may be related to retinoschisis at the site of retinal vessels. In eyes with both cysts and microfolds, retinoschisis at retinal vessels was detected at a much higher rate than eyes with cysts alone.^14^

### Paravascular Lamellar Holes

**Figure 13:**
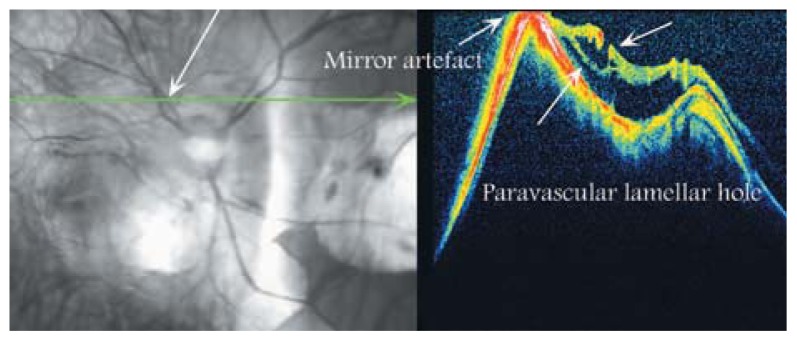
A large paravascular lamellar hole in a pathologic myopic eye. At the right side of the hole, an area of peripapillary detachment is notable.

**Figure 14:**
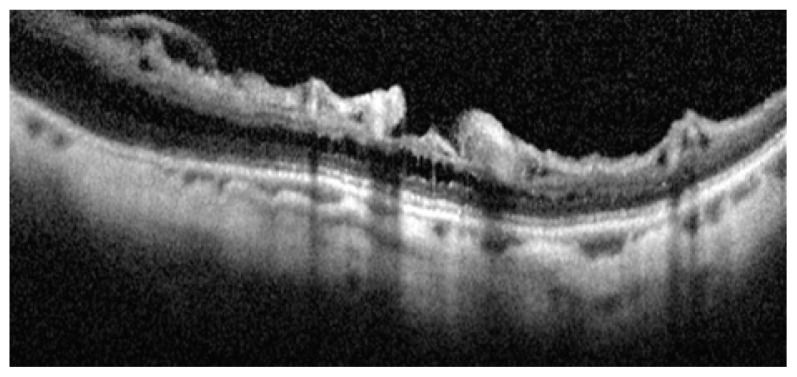
A paravascular lamellar hole which may be due to unroofed paravascular cysts. Retinal schisis is also noted underneath.

The pathogenesis of paravascular lamellar holes has not been elucidated. OCT scans across the superior and inferior vascular arcades can detect such lesions adjacent to paravascular retinal cysts. It seems that detachment of the posterior hyaloid or rupture of the inner wall of paravascular retinal cysts are responsible for creation of paravascular lamellar holes and that paravascular retinal cysts are the precursors of lamellar holes along the vascular arcades as seen in Figures 13 and 14. Paravascular lamellar holes are seen in 26.8% of highly myopic eyes.^14^

Paravascular retinoschisis was frequently associated with paravascular lamellar holes (74%). Remarkably, the incidence of internal limiting membrane (ILM) detachment and macular retinoschisis was significantly higher in eyes with paravascular lamellar holes than those with other paravascular abnormalities. Paravascular lamellar holes were associated with ILM detachment in 44% and with macular retinoschisis in 20% of eyes. More than 80% of eyes with macular retinoschisis had paravascular lamellar holes.^14^ Therefore, paravascular lamellar holes might be an important causative factor for macular retinoschisis.

### Tractional Internal Limiting Membrane Detachment

**Figure 15:**
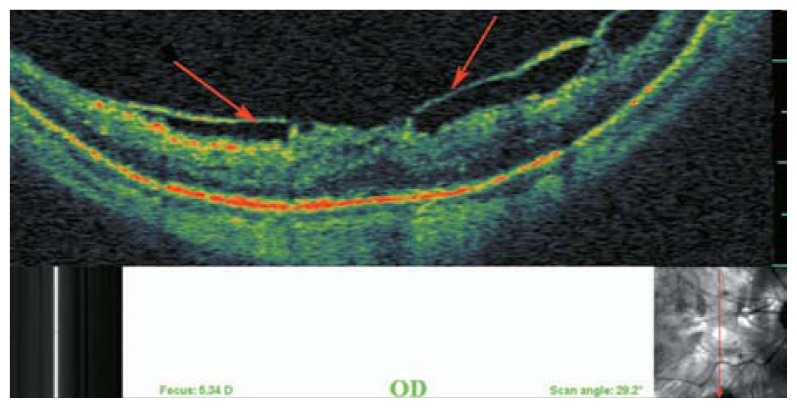
Longitudinal optical coherence tomography crossing the central fovea. It seems that condensed posterior cortical vitreous and incomplete separation from inner retinal layers (red arrows) are responsible for tractional foveal thickening and early schisis.

**Figure 16:**
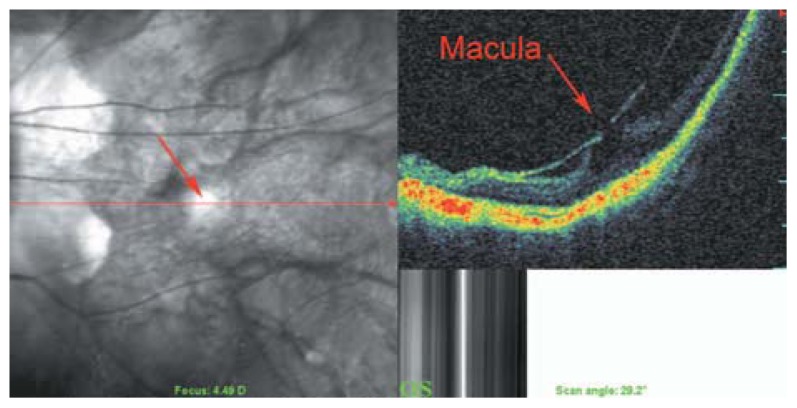
Longitudinal optical coherence tomography in a myopic eye shows more advanced tractional maculopathy resulting in separation of the inner foveal layer and lamellar macular hole formation.

**Figure 17:**
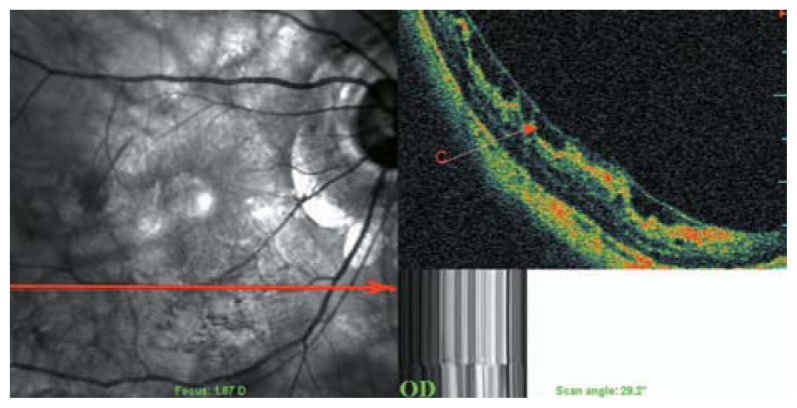
Optical coherence tomography of the inferior macula in a myopic eye shows tractional internal limiting membrane detachment which bridges over inner retinal layers. In this example it resembles a taut cortical vitreous which is not able to expand with the outer parts of the retina.

**Figure 18:**
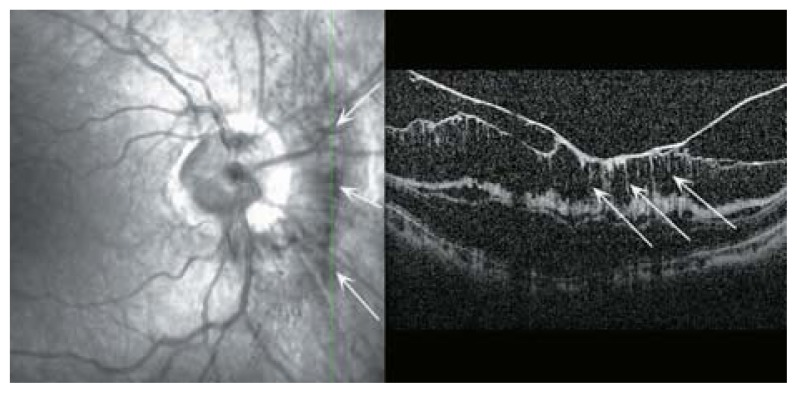
Differentiation between dense cortical vitreous or epiretinal membrane and tractional internal limiting membrane detachment.

Tractional ILM detachment was reported in 2.4%–6% of highly myopic persons.^4^,^17^ Differentiation of ILM detachment from epiretinal membrane is based on the presence of columns that bridge the membrane to the retinal surface. It seems that tractional myopic maculopathy described by Panozzo et al^18^ in 2004 is a variation of longstanding tractional ILM detachment with loss of column bridges due to extreme traction and retinal atrophy. The nature of such tractional components in highly myopic eyes remains unknown but may be due to incomplete posterior vitreous detachment (PVD) and the multilayered nature of the cortical vitreous with abnormally tight attachment to the inner retina. In 67% of patients with ILM detachment, myopic foveoschisis (MFS) was detected^17^, therefore this phenomenon may be an important contributor to separation of the inner layers of the neural retina resulting in macular retinoschisis (Figures 15, 16). Distinction between ILM detachment and incomplete PVD on OCT figures may be very difficult or impossible (Figures 17, 18).

**Figure 19:**
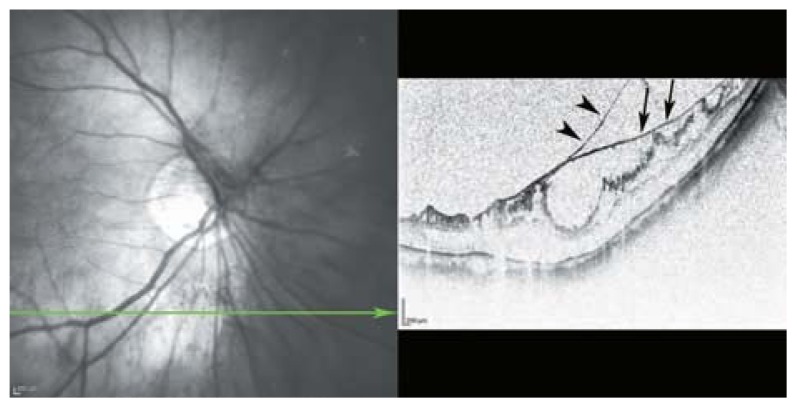
This figure clearly shows the multilayered nature of the posterior cortical vitreous and its tight attachment to the internal limiting membrane (ILM) in myopic eyes (arrow heads) as it merges with the ILM (arrows).

In a study by Bando et al^19^ collagen fibers and cell debris were identified on the inner surface of the ILM peeled from eyes with MFS in 70% of cases. The origin of fibroglial cells identified on the ILM was not clearly determined; however some cell types such as astrocytes, which exist abundantly around retinal vessels, might migrate from the retina through small pores of the inner retina in eyes with paravascular lamellar holes. Migrated cells might then produce collagen fibers and initiate a proliferative response on the ILM, identified as an ILM detachment on OCT. The rigid ILM might prevent the retina from stretching to adjust with the contour of the posterior staphyloma, and contribute to development of macular retinoschisis.^19^,^20^ Tight attachment of the ILM to the posterior cortical vitreous may be responsible for difficult differentiation between them on cross-sectional OCT images (Fig. 19).

### Myopic Foveoschisis

**Figure 20:**
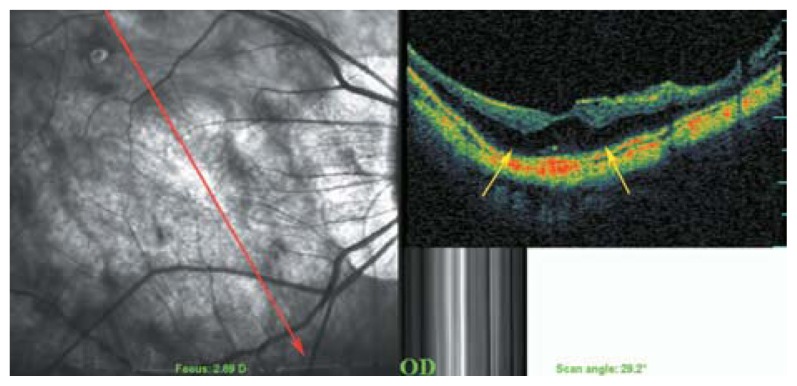
Early myopic foveoschisis may demonstrate as outer retinal layer thickening (yellow arrows) of low reflectivity.

**Figure 21:**
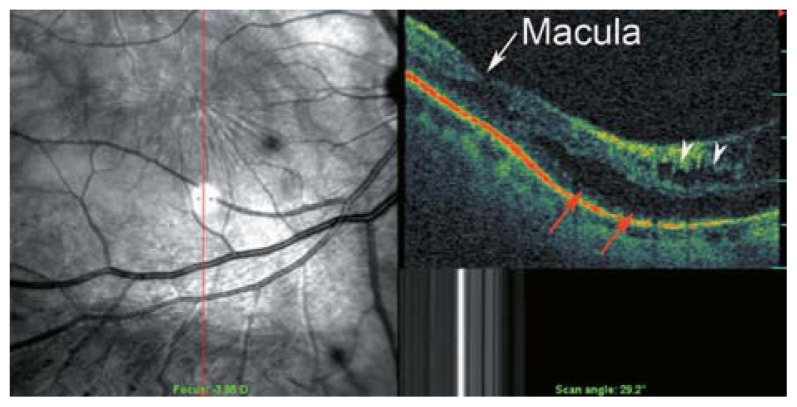
This image shows outer macular schisis (red arrows) accompanied by internal limiting membrane detachment (white arrow heads) extending to the fovea (white arrow).

The prevalence of myopic foveoschisis (MFS) ranges from 9% to 34% in highly myopic eyes with posterior staphylomas.^4^,^21^,^22^ MFS is defined as a separation of intraretinal layers, predominantly outer layers, with subsequent retinal destruction (Figures 20, 21). Potent retinal arteriolar traction is a possible etiology of myopic foveoschisis.^23^

MFS tends to occur in eyes with severe myopic retinopathy.^22^ This sight threatening complication is associated with axial lengthening, chorioretinal atrophy and vitreoretinal interface traction which may be accompanied by both intraocular and outer ocular wall factors (axial length, macular chorioretinal atrophy).^24^ Sometimes it is associated with inner/outer segment defects and this may predict postoperative visual recovery.^25^

**Figure 22:**
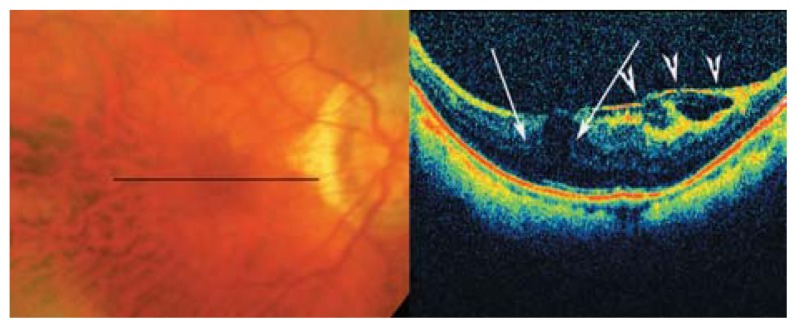
Color fundus photographs of a high myopic patient with mild foveoschisis. The longitudinal B-scan OCT view reveals mild outer retinal layer dehiscence in the fovea (white arrows) with tractional epiretinal membrane, ILM detachment or condense posterior vitreous (arrow heads) above it which may be responsible for these changes.

**Figure 23:**
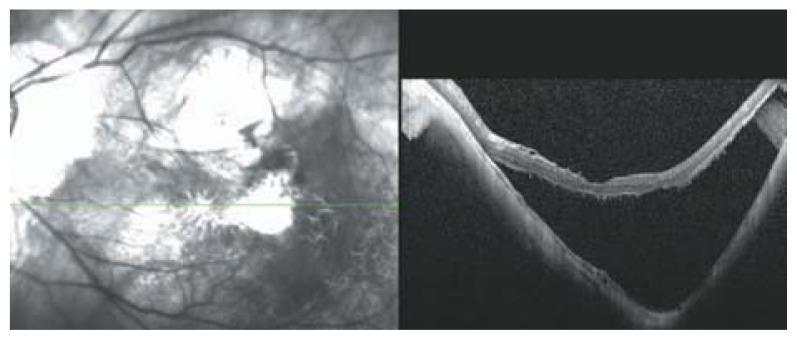
Posterior retinal detachment in a highly myopic eye without macular hole. Detached neurosensory retina shows a corrugated pattern at its outer surface which may be due to lack of outer segment phagocytosis by the retinal pigmented epithelium layer, resulting in outer segment elongation.

**Figure 24:**
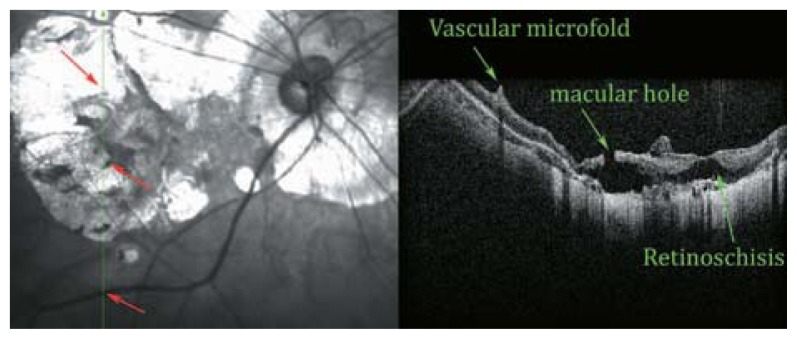
SLO-OCT image of a myopic posterior pole with chorioretinal atrophy. Extreme retinal atrophy, posterior staphyloma, vascular microfolds, and parafoveal retinoschisis result in macular hole formation, and a small localized posterior retinal detachment are present.

Half of patients with MFS have been reported to develop retinal detachment or macular holes within two or more years of follow-up.^26^ Therefore, serial OCT examinations should be performed in these patients. This complication may be related to the presence of vitreoretinal traction (Fig. 22). It seems that macular hole formation is the result of vitreoretinal traction on MFS, but in the absence of vitreoretinal traction, posterior retinal detachment without macular hole may also occur, which seems to be related to staphyloma and inward forces exerted by rigid and taut retinal vessels and ILM (Fig. 23). Sometimes inherent weakness of the fovea due to schisis with vitreoretinal traction contributes to development of a macular hole (Fig. 24).^26^

### Choroidal Neovascular Membrane

**Figure 25:**
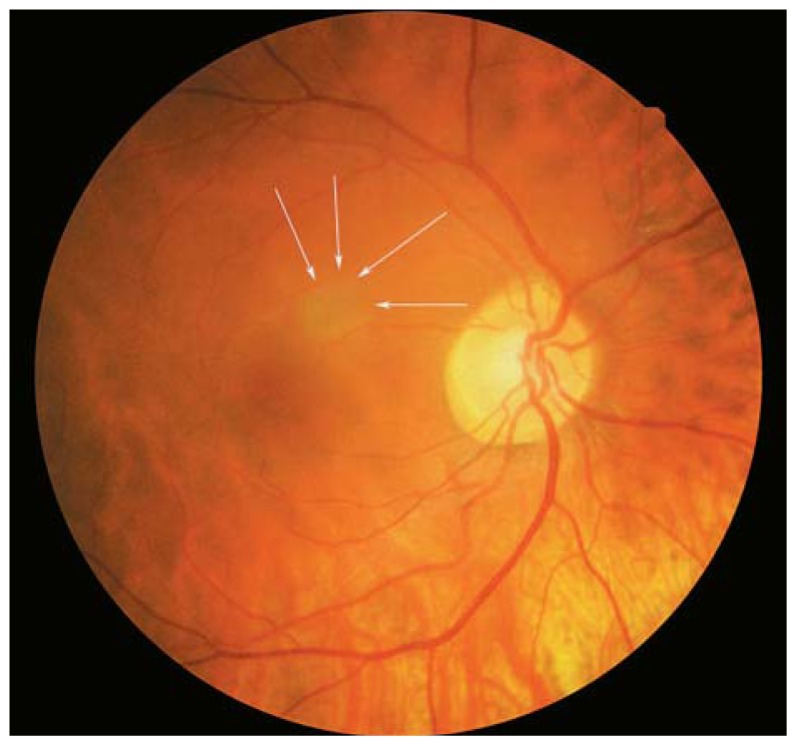
Color fundus photograph of a myopic eye with decreased vision of a few weeks’ duration due to choroidal neovascularization. A grey elevated mass is noted in the parafoveal area (arrows).

**Figure 26:**
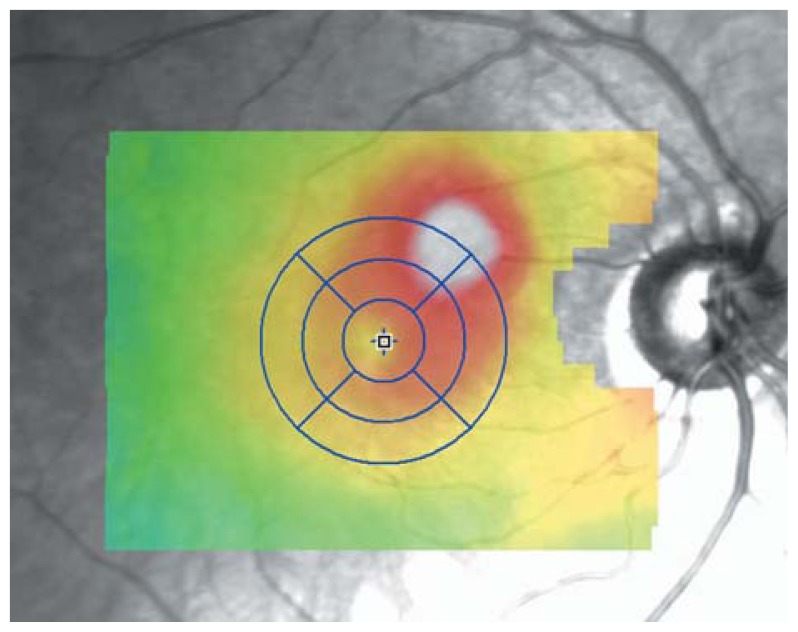
Retinal topographic map by optical coherence tomography reveals localized retinal elevation at the area of neovascularization.

**Figure 27:**
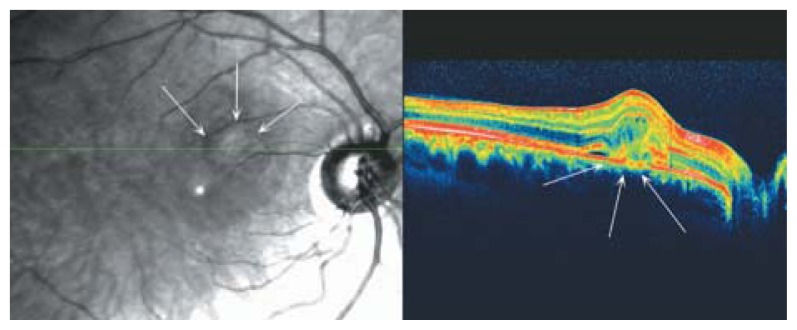
SLO-OCT examination of that area reveals an active neovascular growth under the retina which blends with outer retinal layers and results in mild disorganization of that area. The amount of subretinal fluid collection is minimal.

**Figure 28:**
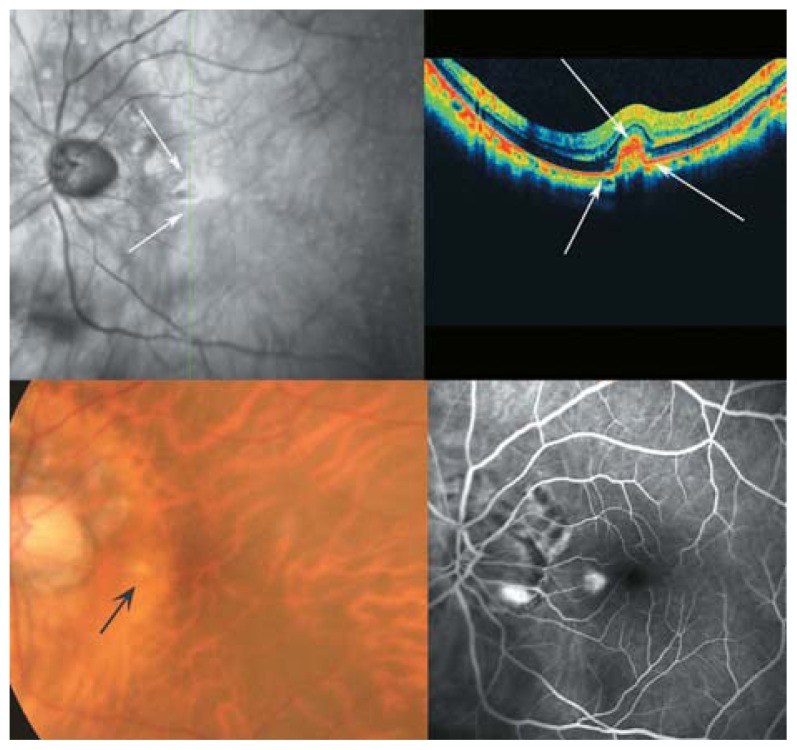
Another example of choroidal neovascularization in the extrafoveal area of a highly myopic patient (optical coherence tomography and fluorescein angiography). The inner surface of the active neovascular tuft is feathery with medium reflectivity. Evolution to inactivation and scar formation is accompanied by increased reflectivity and sharpness of the edges of the lesion.

**Figure 29:**
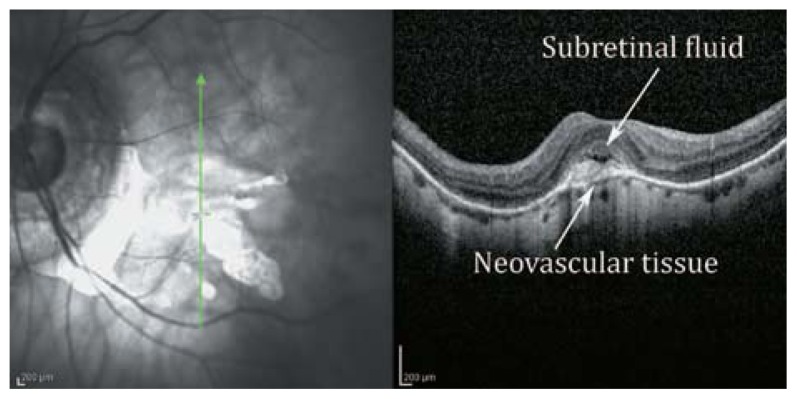
Another example of active myopic choroidal neovascularization with a neovascular membrane under the fovea together with subretinal fluid collection and destruction of underlying Bruch’s membrane (white lower arrow).

Choroidal neovascular membrane (CNV) is considered to be the most important sight-threatening complication of high myopia. Myopic CNV has been classified into active, scar and atrophic stages.^27^ In the active stage, OCT displays the neovascular tuft as a highly reflective dome-shape elevation above the RPE. No considerable subretinal fluid accumulation around the lesion is identified (Figures 25–29).

In the scar stage, only the surface of the neovascular tuft shows high reflectivity and the tissue underneath is markedly attenuated. Overlying retinal layers are not disarranged or disorganized, cystic changes are also absent.

**Figure 30:**
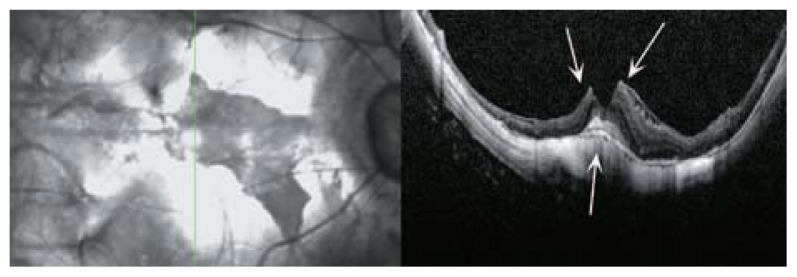
End-stage neovascular membrane with scar formation and lamellar macular hole which may be the result of tractional internal limiting membrane detachment or the consequence of inactivation of neovascular membrane. Macular hole in this case (white arrows) may be the result of atrophic changes after the occurrence of subfoveal choroidal neovascularization.

In the atrophic stage, the CNV becomes flat and chorioretinal atrophy around the regressed CNV is highly reflective.^27^ Eyes in the atrophic stage have a higher risk of developing a macular hole (14%) (Fig. 30).^28^ Neovascular lesions in myopic eyes are associated with considerably less retinal edema and subretinal and intraretinal fluid as compared to their counterparts in the setting of age-related macular degeneration; furthermore, pigmented epithelial detachments almost never exist.^29^

Macular holes always exist at the edge of a pigmented lesion suggestive of an old CNV. Therefore periodic OCT examinations for evaluation of macular holes or macular retinoschisis, even in patients in atrophic stages, are recommended. The retina in the area of chorioretinal atrophy around the CNV is very thin regardless of coexisting macular retinoschisis, but the retina at the edge of CNV in myopic eyes with large chorioretinal atrophy (larger than 1 disc diameter) has been reported to be significantly thinner in eyes with chorioretinal atrophy smaller than 1 disc diameter around the CNV.^28^

### Posterior Retinal Detachment

**Figure 31:**
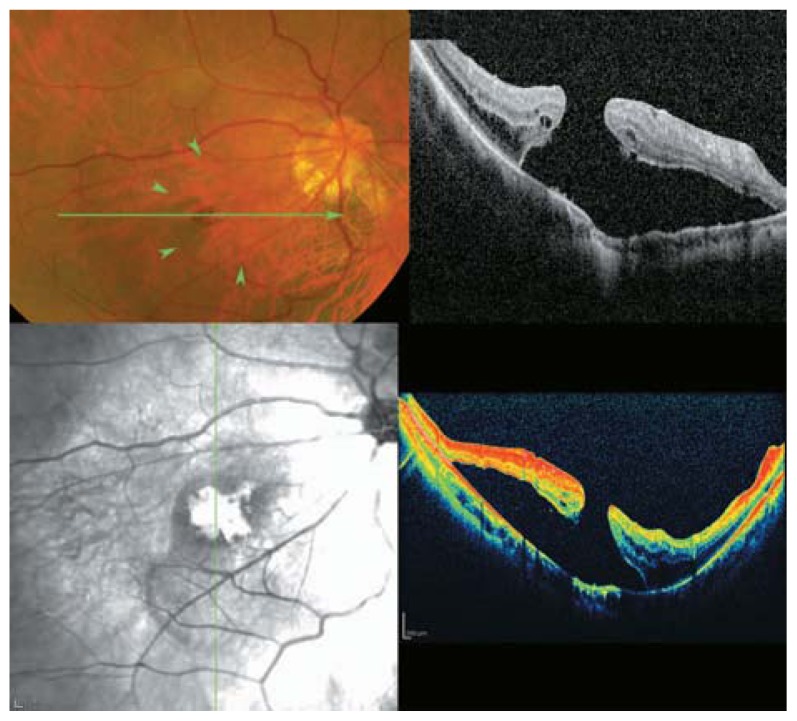
SLO, optical coherence tomography (OCT) and color fundus photography of a highly myopic eye with posterior retinal detachment and a full thickness macular hole. The detachment is limited to the posterior pole which is clearly evident on OCT. Multiple cystic spaces at the edges of the hole indicate chronicity of the detachment. The margins of the detachment are shown by green arrowheads in the color fundus photograph.

Posterior retinal detachment is another consequence of ocular expansion and posterior staphyloma in high myopic eyes. Full thickness macular hole and tractional maculopathy may accompany this finding and may be its contributor (Fig. 31).

### Macular Hole

**Figure 32:**
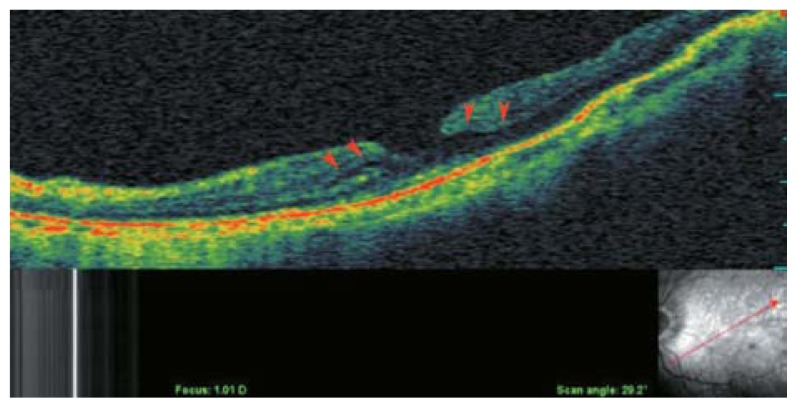
An early lamellar macular hole due to expansion of the posterior pole. Clefts in the middle retinal layer (red arrow heads) are the signs of early schisis or lamellar hole formation.

**Figure 33:**
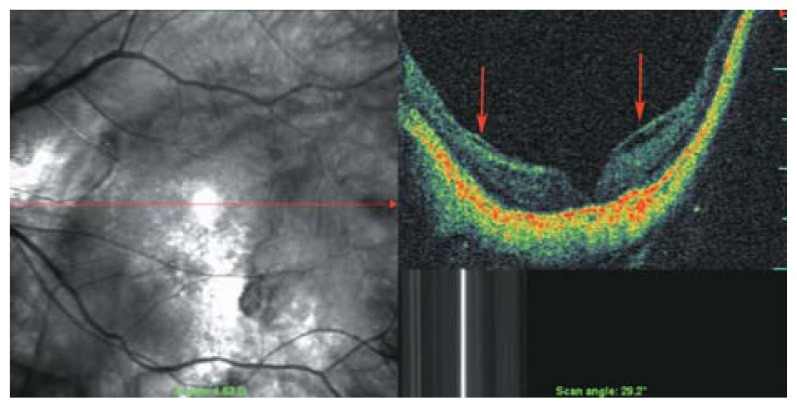
Tractional macular band (red arrows) may contribute to the central foveal cleft (deep lamellar macular hole) in this SLO-optical coherence tomography.

**Figure 34:**
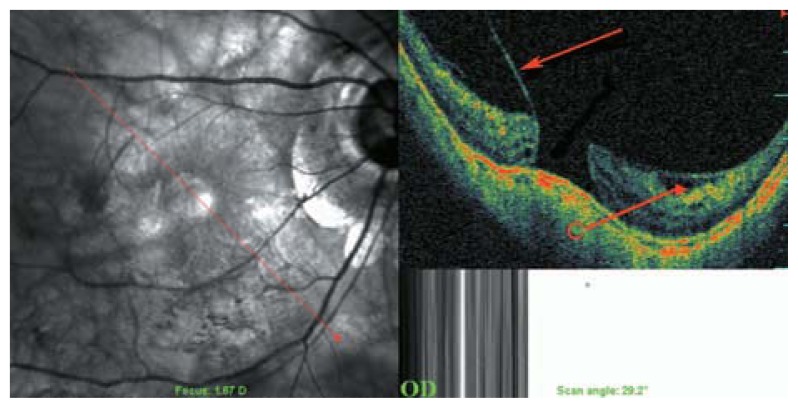
Full-thickness macular hole which may be due to tractional retinal components that separate central foveal tissues (red arrows).

**Figure 35:**
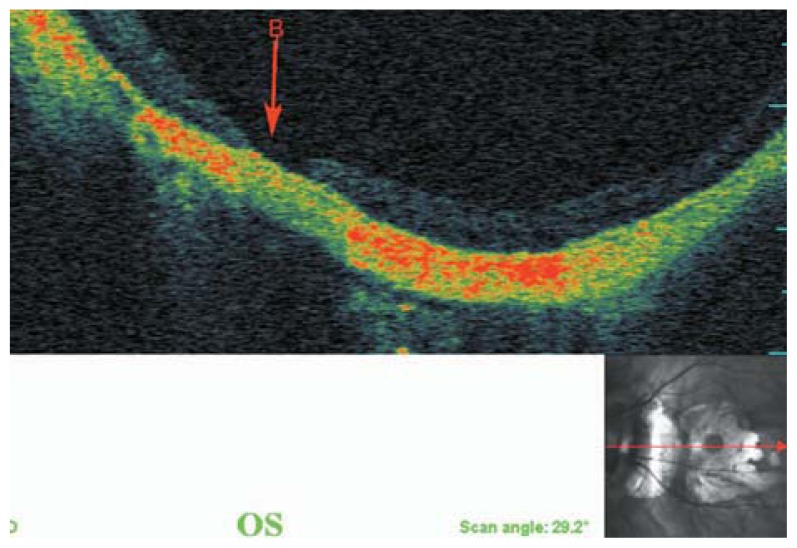
Full-thickness macular hole (red arrow) which may be due to globe expansion separating retinal tissues in the fovea.

Macular hole formation in myopic eyes has multiple etiologies. Expansion of the globe, tractional components and growth of neovascular membranes through clefts in the posterior pole may create partial thickness and/ or full-thickness macular holes. In asymptomatic myopic persons, macular holes were detected in 6.26% of cases.^30^ Macular holes in myopic eyes have multiple forms and multiple etiologies (Figures 32–35).

### Other Changes

**Figure 36:**
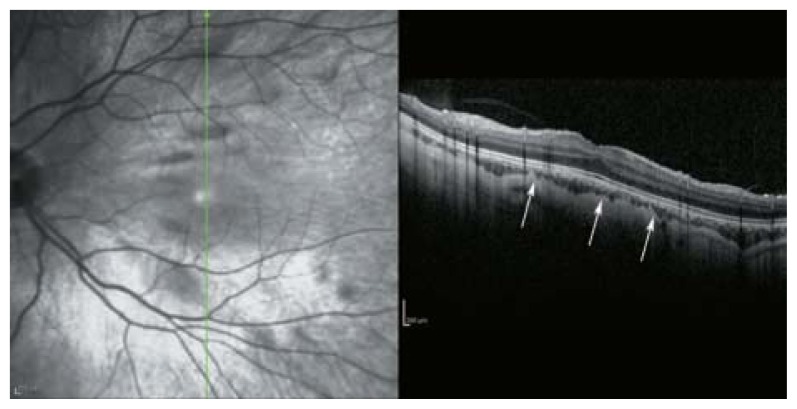
Due to vertical scan orientation of the longitudinal optical coherence tomography, the posterior staphyloma cannot be appreciated, but choroidal thinning (to about half-normal thickness) is visible (white arrows).

**Figure 37:**
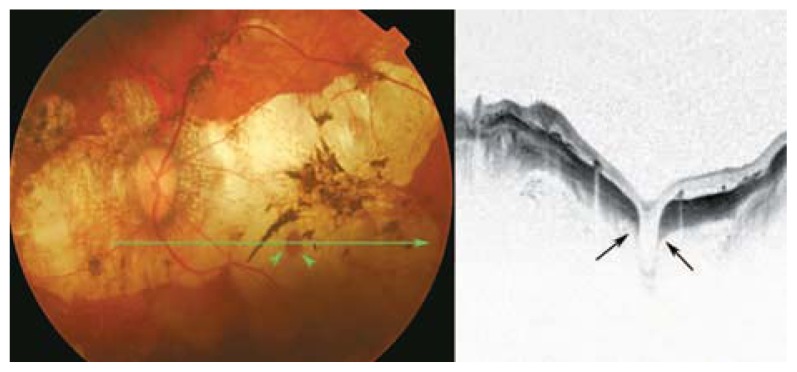
Due to globe expansion in pathologic myopia, apertures of the sclera which are perforated by ciliary vessels are visible (black arrows) as large-diameter, full-thickness scleral defects together with outward displacement of the retina. This is usually accompanied by retinal pigmented epithelium/choroidal atrophy.

Pathologic myopia is accompanied by thinning and stretching of the globe. With increasing age and myopia, these changes further progress.^31^ Therefore, choroidal thinning and visibility of the sclera is another OCT finding in severe myopia (Figures 36, 37).

## CONCLUSION

Individuals with high myopia are subject to various retinal pathologies including posterior pole and peripapillary lesions. Because of extreme retinal thinning and chorioretinal changes in these persons, simple fundus examination may miss subtle retinal pathologies. OCT is an accurate tool which can localize such anatomical changes. It seems to be the paraclinical test of choice for highly myopic eyes.
